# LDAcoop: Integrating non‐linear population dynamics into the analysis of clonogenic growth *in vitro*

**DOI:** 10.1002/1878-0261.70185

**Published:** 2025-12-26

**Authors:** Nikko Brix, Daniel Samaga, Katharina Gehr, Benedek Dankó, Mohamed Schumann, Guido Drexler, Ahmed Alnatsha, Georg Beyer, Ujjwal Mahajan, Martin Selmansberger, Julia Mayerle, Claus Belka, Horst Zitzelsberger, Kirsten Lauber

**Affiliations:** ^1^ Department of Radiation Oncology University Hospital, LMU München Munich Germany; ^2^ Research Unit Translational Metabolic Oncology (TMO), Institute for Diabetes and Cancer (IDC) Helmholtz Diabetes Center, Helmholtz Munich Neuherberg Germany; ^3^ Joint Heidelberg‐IDC Translational Diabetes Program, Department of Inner Medicine I Heidelberg University Hospital Heidelberg Germany; ^4^ German Center for Diabetes Research (DZD) Neuherberg Germany; ^5^ Bavarian Center for Cancer Research (BZKF) partner site Munich Munich Germany; ^6^ Department of Internal Medicine II University Hospital, LMU München Munich Germany; ^7^ Department of Pharmacology and Toxicology National Institute of Pharmaceutical Education and Research (NIPER) Mohali India; ^8^ German Cancer Consortium (DKTK) partner site Munich Munich Germany

**Keywords:** Allee effect, cellular competition, cellular cooperation, clonogenic growth, clonogenic survival, limiting dilution assay

## Abstract

The limiting dilution assay (LDA) is a key method to quantify clonogenic cells with self‐renewing capacity *in vitro*, crucial for preclinical cancer research and therapy response assessment. It estimates the frequency of individual clonogenic, stem‐like cells within a population based on their ability to form colonies with ≥50 cells at limiting cell numbers. Standard LDA analysis relies on linear, single‐hit Poisson models, yet clonogenic growth under single‐cell conditions often involves cooperative or competitive dynamics, violating this linearity assumption. Here, we present a modeling framework incorporating non‐linear population dynamics into LDA analysis and introduce LDAcoop, an R‐based tool for universal quantification of clonogenic cells in LDA formats. Across multiple cancer cell types, we benchmarked LDA against the colony formation assay (CFA) and show that LDA outperforms CFA, especially for patient‐derived organoids, suspension cultures, and higher throughput applications. This renders the LDA format particularly suitable for larger‐scale pharmacogenomic screening and drug sensitivity testing in complex models. Our results establish LDA and LDAcoop as versatile, scalable tools for robust quantification of clonogenic growth, supporting preclinical drug development and molecular precision oncology research.

## Graphical abstract

Limiting dilution assays (LDAs) quantify clonogenic growth by seeding serial dilutions of cells and scoring wells for colony formation. The fraction of negative wells is plotted against cells seeded and analyzed using the non‐linear modeling of LDAcoop. LDAs perform comparably to colony formation assays (CFAs) but require less scoring time and are better suited for suspension cells, 3D cultures, and higher throughput formats.

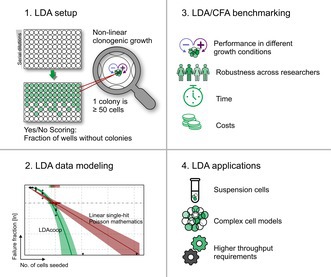

**Abbreviations**
C: colony count per well
CFA: colony formation assay
DPP: decapentaplegic
F: failure fraction
FBS: fetal bovine serum
HNSCC: head and neck squamous cell carcinoma
LDA: limiting dilution assay
OR: odds ratio
PDAC: pancreatic ductal adenocarcinoma
ROCK: Rho‐associated protein kinase
S: number of seeded cells per well
SFs: surviving fractions
SHPM: single‐hit Poisson model
WNT: wingless/integrated

## 1 Introduction

The quantification of clonogenic cancer cells *in vitro* represents a key method in the toolbox of preclinical cancer research and its subdisciplines, including basic tumor biology, molecular precision oncology, immuno‐oncology, and preclinical therapy development. In this context, clonogenic growth describes the capacity of single cells to form colonies through self‐renewal and division. Clusters with a minimum of 50 cells which developed from one singular ancestor cell are conventionally considered as a surrogate for infinite growth or—at least—the capacity for multiple rounds of cell division, respectively [1, 2].

**Fig. 1:**
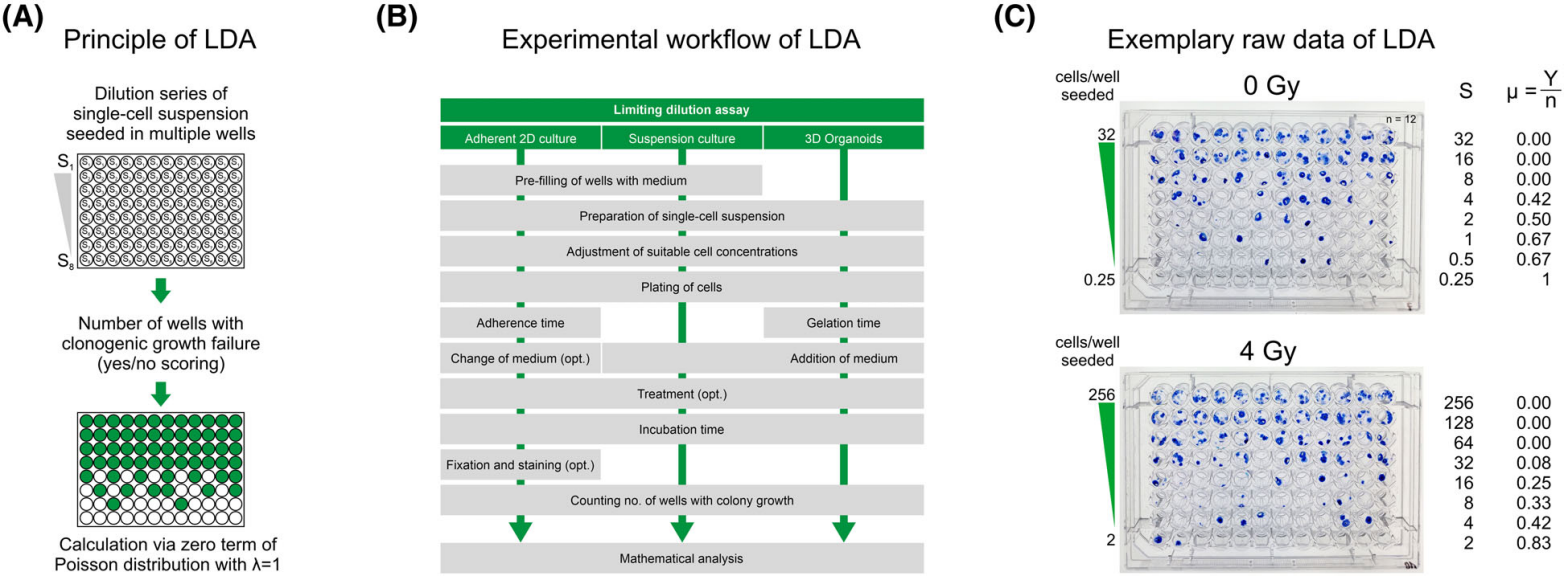
The limiting dilution assay (LDA). (A) Principle of the LDA. A dilution series of the single‐cell suspension of interest (with cell numbers *S*_1_ to *S*_8_) is seeded into a multi‐well plate with multiple replicates per dilution. After an appropriate incubation time sufficient to allow at least 6 cell doublings, the number of wells with clonogenic growth failure is determined, and clonogenic activity is calculated via the zero term of the Poisson distribution with *λ* = 1. (B) Experimental workflow as performed in this manuscript. (C) Examples of raw data from MCF7 cells. LDA plates were seeded in an 8‐step geometric dilution series with 12 replicates per dilution. The numbers of seeded cells (*S*) and the fractions of wells with clonogenic growth failure (*μ = Y*/*n*) are indicated.

**Fig. 2:**
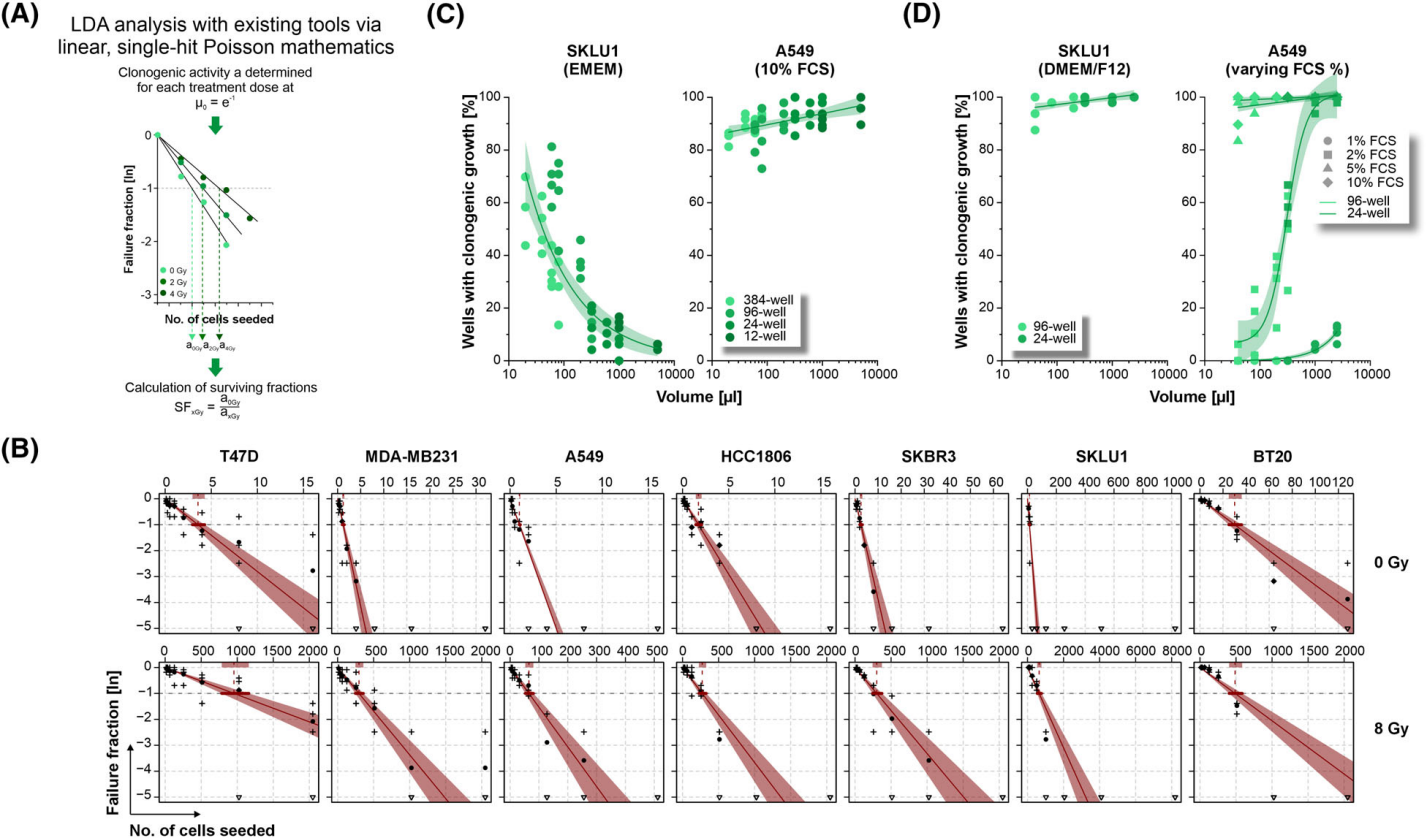
Linear single‐hit models do not account for cooperative and competitive clonogenicity and fail to robustly describe clonogenic growth behavior of several cell lines in the LDA format. (A) Workflow of LDA data analysis with existing tools relying on linear, single‐hit Poisson mathematics (such as https://bioinf.wehi.edu.au/software/elda/ [3]). (B) Examples of LDA raw data sets of seven different cancer cell lines with linear regression superimposed in red (95% confidence intervals are displayed in pale red). Data of each cell line were generated in three to four independent biological replicates. Mean ln(*μ*) values are represented by filled dots (•). + and ▽ symbols represent data points from individual replicates. (C) Analysis of mammalian single‐cell growth behavior types. Identical cell numbers of SKLU1 and A549 single‐cell suspensions (10 cells/well for SKLU1, 3 cells/well for A549) were seeded into 384‐well, 96‐well, 24‐well, and 12‐well plates in culture medium volumes of 20–5000 μL per well, and the percentage of wells with clonogenic growth was determined after 21 days for SKLU1 and 14 days for A549 cells. Data of four independent biological replicates each performed in 48–96 technical replicates are given, and power regression lines with 95% confidence intervals are superimposed. (D) Identical cell numbers of SKLU1 and A459 cells were seeded as in (C) in varying culture medium volumes of DMEM/F12 medium supplemented with 10% FCS (left panel) or varying concentrations of FCS (right panel). Data of four independent biological replicates each performed in 48–96 technical replicates are given, and regression lines (power or logistic regressions) with 95% confidence intervals are superimposed.

A widely used technique to determine the frequency of cancer cells with self‐renewing, clonogenic potential is the limiting dilution assay (LDA) [3]. Serial dilutions of single‐cell suspensions are seeded into multi‐well plates, and the fraction of wells with clonogenic growth for each seeded single‐cell number is determined (Fig. 1). The frequency of clonogenically active cells in the single‐cell population can be inferred from the failure fraction, that is, the fraction of wells without clonogenic growth, according to the zero term of the Poisson distribution at an expectation of one [3] (Fig. 2A). A similarly well‐established technique to quantify clonogenic growth is the colony formation assay (CFA) [1, 4]. Here, the exact colony count per culture dish in relation to the number of seeded cells is determined [1]. As a mutual characteristic, the LDA and the CFA interrogate clonogenic growth behavior at very low densities, often less than one cell per mm^2^ surface area. Under these extreme conditions, colony formation is critically dependent on growth‐supporting soluble factors in the culture medium, and it was not until the 1950s that reproducible mammalian single‐cell growth became technically feasible [5, 6]. At this time, scientists had a thorough understanding of the *in vitro* growth requirements for various types of mammalian normal and cancerous cells, and clonogenic single‐cell growth was known to be dependent on biochemically defined molecules—salts, amino acids, sugars, and cofactors—as well as a complex and undefined mixture of animal‐derived essential components typically contained in serum preparations [7, 8]. Although clonogenic growth of selected cell lines became feasible in fully synthetic, chemically defined media in the 1960s [9], serum preparations or other animal‐derived supplements, such as bovine pituitary extract, are still widely used in contemporary cell culture [10].

In addition to the biochemical composition of the culture medium itself, cell‐derived molecules, such as growth factors, hormones, low‐molecular‐weight metabolites, and growth inhibitors, can influence single‐cell growth via auto‐ and paracrine mechanisms [11], suggesting that phenomena of cellular cooperation and competition influence clonogenic growth patterns to varying extents [12, 13]. In this context, it has been proposed that the absence of cooperating or competing neighboring cells can respectively slow down, inhibit, or enhance cell growth at low plating densities [14, 15, 16, 17, 18]. The reduction in reproductive fitness at limiting cell densities is known as the Allee effect—a concept in ecology to describe a *per capita* population growth rate, which gradually decreases at low population densities [16, 19, 20]. On the contrary, neighboring cells can also exert competitive effects under limiting cell numbers, via deprivation of critical nutrients in the chosen culture medium and/or secretion of growth‐inhibitory factors [18]. Hence, albeit less common than cellular cooperation, competitive mechanisms may contribute to nonlinear clonogenic growth phenomena as well [10, 17, 18, 21], and the net effect of cooperative and competitive effects in a culture dish obviously depends on various parameters, including the cell culture model and the culture conditions chosen.

Cellular cooperation and competition strongly affect the mathematical analysis of clonogenic survival experiments. However, standard data analysis workflows and associated computational tools fail to account for these non‐linear phenomena, causing the linear mathematical models to diverge from the real‐world data they are meant to represent [22]. To address this limitation in the CFA, we recently developed a mathematical modeling approach that incorporates nonlinear cell‐number‐to‐clonogenic‐growth relations and thereby improves analytical reliability and robustness [1, 22]. In this study, we investigated the effects of nonlinear clonogenicity in the LDA format. Considering that clonogenic survival analyses in both the CFA and LDA format are performed using very different dilutions of single‐cell suspensions within one experiment [1, 3], we hypothesized that cooperative as well as competitive effects may similarly lead to unreliable estimates of clonogenic cell frequencies in the LDA format due to violations of the linearity assumption in stable ratios of cell numbers to measures of successful clonogenic growth. Accordingly, we generalized the established linear, single‐hit Poisson model via power law and included a nonlinearity parameter, which accounts for cooperative and competitive clonogenic growth phenomena. To make this workflow accessible for a broad audience, we developed LDAcoop, an R package published on CRAN (accompanied by an online shiny app version thereof) designed to account for nonlinear clonogenic growth patterns in LDA analysis. Finally, we benchmarked the LDA format against the CFA format with regard to experimental procedures, scoring, data output, speed, and versatility in different cancer cell culture systems.

## 2 Materials and methods

### 2.1 Development of LDAcoop

#### 2.1.1 Statistical modeling of nonlinear clonogenic growth behavior

Let *n* ∈ N, *n* > 0 be a number of wells with an identical number of cells. Each well is seeded with an expected number of cells *S* ∈ N, *S* > 0. The probability of failure (no growth) in each well is identical and independent for all wells and denoted with *μ* ∈ [0, 1]. The number of failures *Y* is therefore a binomially distributed random variable:

PY=r=nrμr1−μn−r

The probability of success (1−μ) on the level of wells, in turn, depends for each well on the expected number of cells seeded *S*. Assuming an identical and independent probability of growth *p* ∈ [0, 1] for every cell seeded, we find as known from the single‐hit Poisson model (SHPM) an expectation of

$$\lambda_{\text{SHPM}}=p\cdot S$$

‘active’ cells in a well. As a generalization of this linear approach and analogously to modeling non‐linear growth behavior in CFA analysis, the deviation from linearity in mean activity is accounted for by introducing a non‐linearity coefficient *b* > 0. Specifically, 0 < *b* < 1 corresponds to competitive, *b* = 1 to linear, and *b* > 1 to cooperative growth. This yields a power law relation

$$\lambda =p\cdot S^{b}$$

Given a mean activity *λ* > 0, the number of active cells in a well *X* ∈ *N*_0_ follows approximately the Poisson distribution

$$P_{\lambda }\left(X=k\right)=\frac{\lambda^{k}}{k!}e^{-\lambda }$$

The probability of failure is therefore:

$$\mu =P_{\lambda }\left(X=0\right)=e^{-\lambda }=e^{-p\cdot S^{b}}$$

and with $\alpha =\ln\left(p\right)$ we find

$$\ln\left(-\ln\left(\mu \right)\right)=\alpha +b\cdot \ln\left(S\right)$$

For *m* sets of wells with varying numbers of cells *S*_*i*_ and corresponding observations $\mu_{i},i\in \left\{1,\;..,m\right\}$ this is a generalized linear regression model of the binomial family with log–log link function. Model fitting is conducted using statistical software R (*glm* function from *stats* package).

#### 2.1.2 Definition of clonogenic activity

We define the clonogenic activity *a* as the number of cells that need to be seeded to achieve on average 1 colony per well, resulting in $e^{-1}$ (i.e., approximately 37%) wells with exactly 1 colony and $e^{-1}$ wells with growth failure:

$$a=e^{\frac{-\alpha }{b}}={\left(p^{-1}\right)}^{\frac{1}{b}}$$

For cells with linear clonogenicity, the clonogenic activity equals the inverse of the active cell frequency [3]. A cell frequency suggests an easy and straightforward interpretation: ‘each cell has a certain probability of having some property’, and this probability is the same for all cells, independently of the others. However, the chance of forming a new colony also depends on the surroundings, particularly the number of neighboring cells and their secretomes. Against this background, quantification through an active cell frequency is misleading in the context of cellular cooperation or competition, and the definition of clonogenic activity focuses directly on this total number of cells, which provides together the activity of interest.

The uncertainty of the clonogenic activity is calculated from the confidence bands of the model fit with statistical software (R statistical software, *predict.glm* function from basic *stats* package). Cell numbers that result in upper or lower bounds of 37% negative wells are the lower and upper bounds of the confidence interval of the clonogenic activity (Fig. S1). Alternatively, the uncertainty can be approximated via conventional error propagation (e.g., through first‐order Taylor series approximation) in terms of standard deviation.

#### 2.1.3 Calculation of surviving fractions

Changes in the clonogenic activity under treatment (e.g., upon exposure to ionizing radiation) are quantified using the survival fraction *SF*. In this regard, the term survival refers to the retention of clonogenic capacity following treatment. Accordingly, the survival fraction *SF* is defined as the ratio of the number of cells required to result in an identical expectation of the same reference activity (e.g., 37% failure rate) without and with treatment.

Let $\alpha_{0}$ and $b_{0}$ denote the model parameters fitted to the untreated cells, and $\alpha_{t}$ and $b_{t}$ the parameters for the treated cells. The survival fractions are calculated as

$${\mathrm{SF}}_{t}=\frac{a_{0}}{a_{t}}=\frac{e^{\frac{-\alpha_{0}}{b_{0}}}}{e^{\frac{-\alpha_{t}}{b_{t}}}}=e^{\frac{\alpha_{t}}{b_{t}}-\frac{\alpha_{0}}{b_{0}}}$$

Uncertainties of the survival fractions can be approximated via conventional error propagation (first‐order Taylor series). As a more robust approach, we approximate SF 95% confidence intervals by combining 83.5% confidence intervals of the corresponding clonogenic activities [23, 24, 25], yielding an overall coverage close to 95% when propagated (explicit formulas are provided in Fig. S1). Therefore, given a reference clonogenic activity of $a_{0}$ with associated 83.5% confidence interval $\left[a_{0,\mathrm{lb}},\;a_{0,\mathrm{ub}}\right]$ and a clonogenic activity $a_{t}$ of a treated group with associated confidence interval $\left[a_{t,\mathrm{lb}},\;a_{t,\mathrm{ub}}\right]$, the 95% uncertainty interval of the survival fraction is approximated as $\left[\;\frac{a_{0,\mathrm{lb}}}{a_{t,\mathrm{ub}}},\;\frac{a_{0,\mathrm{ub}}}{a_{t,\mathrm{lb}}}\right]$ (Fig. S1).

#### 2.1.4 Utilization of biological replicates

Individual biological replicates vary in their characteristics. Thus, measurements from the same biological replicate are not fully independent. Still, there is no unique model including this variability, but multiple options exist how this or that aspect of inter‐replicate variability could be modeled. Therefore, to assess inter‐replicate variability, we strongly recommend indicating individual biological replicates in plots of the clonogenic activity. This offers direct and easy visual inspection of the magnitude of inter‐replicate variability.

In case the inter‐replicate variability is considered rather small with respect to its intrinsic stochasticity, combining the number of wells before modeling is reasonable (i.e., numbers of wells from biological replicates are added up). The intrinsic stochasticity stems from the binomial characteristic of the number of responding wells. As an example, the 95% expectation range for 12 wells with probability of nonresponding *p* = 1/3 is 1 to 7 nonresponding wells (which on ln(μ)‐scale corresponds to a range of −2.5 to −0.5).

In case inter‐replicate variability is considered high and serious underestimation of the calculated uncertainties is expected, all replicates are to be analyzed separately. This means clonogenic activities and survival fractions are calculated for the replicates separately. In that case, overall clonogenic activities and associated uncertainties can be derived from the sets of individual clonogenic activities and survival fractions by calculating the mean and standard error of the mean.

In case of low numbers of biological replicates combined with extremely high numbers of wells per biological replicate, the calculated uncertainties will also be underestimated (as in the case of high inter‐replicate variability). In this case, the experiments should be separated by replicate as well.

### 2.2 Statistical methods

All calculations were performed in R version 4.2.0 (2022‐04‐22; R Core Team. R: A Language and Environment for Statistical Computing. R Foundation for Statistical Computing, Vienna, Austria, 2022). LDA data was analyzed by CRAN LDAcoop package (https://cran.r‐project.org/web/packages/LDAcoop/index.html). A shiny app version of LDAcoop is available under https://helmholtz‐munich‐zyto.shinyapps.io/LDAcoop/. For comparison, in Fig. 2B, LDA data (number of cells per well, number of wells, number of positive wells) were also fitted with the ‘ELDA’ function of the ‘statmod’ R package [3]. Improvement in model fit was formally assessed by likelihood‐ratio tests (LRT) on deviance differences between nested models, and model adequacy was additionally evaluated using Akaike's information criterion (AIC).

### 2.3 2D cell culture

The breast cancer cell lines T47D (RRID:CVCL_0553), SKBR3 (RRID:CVCL_0033), BT20 (RRID:CVCL_0178), MDA‐MB231 (RRID:CVCL_0062), HCC1806 (RRID:CVCL_1258), and DU4475 (RRID:CVCL_1183), and the lung cancer cell lines A549 (RRID:CVCL_0023) and SKLU1 (RRID:CVCL_0629) were purchased from either ATCC (Manassas, VA, USA), CLS (Heidelberg, Germany), or DSMZ (Braunschweig, Germany). Cells were cultivated in a humidified atmosphere at 37 °C in culture media (all from Thermo Fisher Scientific, Schwerte, Germany) supplemented with fetal bovine serum (FBS), 100 U·mL^−1^ penicillin, and 0.1 mg·mL^−1^ streptomycin. Details are listed in Table S1. Cell line authentication was performed via short tandem repeat typing (service provided by DSMZ), and all experiments were performed with cultures within 10 passages of authentication. Additionally, cell cultures were routinely screened to be free from mycoplasma contamination (MycoAlert Detection Kit, Lonza, Basel, Switzerland), and only cultures confirmed to be mycoplasma‐free were included in experiments.

### 2.4 Organoid cell culture

Organoid cell cultures derived from pancreatic ductal adenocarcinoma (PDAC) and head and neck squamous cell carcinoma (HNSCC) were used as previously established [26, 27]. Their original generation was performed with written informed consent from all participants, in accordance with the Declaration of Helsinki, and with approval from the Ethics committee of the LMU Munich Medical Faculty. The details of medium composition are given in Tables S2 and S3. All organoids were passaged weekly, PDAC organoids by mechanical dissociation and HNSCC organoids by enzymatic dissociation using TrypLE™ Express (Thermo Fisher Scientific) and subsequent use of the trypsin inhibitor from *Glycine max*. (Sigma‐Aldrich, Taufkirchen, Germany). Upon dissociation, the rho‐associated protein kinase (ROCK) inhibitor Y‐27632 (Selleckchem, Munich, Germany) was added at 10 μm for 1 week (PDAC), or 1 day (HNSCC), respectively. Short tandem repeat typing and mycoplasma testing were performed as described for 2D cell cultures.

### 2.5 X‐ray treatment

Irradiation was performed using an RS225 X‐ray tube (X‐Strahl, Camberley, UK) operated at 200 kV and 10 mA with an integrated Thoraeus filter (1 Gy in 242 s).

### 2.6 Analysis of clonogenic survival of adherent cell lines by limiting dilution assay and colony formation assay

For the analysis of clonogenic survival of adherent cells in the LDA format, geometric dilution series of single‐cell suspensions were seeded into 96‐well plates (2^−3^–2^12^ cells per well). Upon adherence, the medium was refreshed (200 μL per well), and cells were irradiated with the indicated doses. Clonogenic growth was allowed for 14 to 21 days; cells were fixed and stained using 80% ethanol containing 0.8% methylene blue (Sigma Aldrich, Taufkirchen, Germany). Plates were screened for clonogenic growth at 8–40× magnification. Wells containing at least one colony with ≥50 cells were considered positive. Surviving fractions were calculated using the provided R tool LDAcoop.

CFA format data and power regression‐based results (i.e., the fraction of clonogenic cells per well after a given treatment) were taken from Brix et al. [22].

### 2.7 Limiting dilution assay with suspension cells

Geometric dilutions of DU4475 single‐cell suspensions were seeded into 96‐well plates (2^0^–2^13^ cells per well) and the total volume per well was adjusted to 200 μL in all wells. Then, cells were irradiated and incubated for 23 days. Screening was performed at 40 × magnification without prior staining, and surviving fractions were obtained as described for adherent cells.

### 2.8 Limiting dilution assay with organoids

Single‐cell suspensions of organoids were generated using a mixture of TrypLE™ Express (Thermo Fisher Scientific, Dreieich, Germany), Dispase (2 mg·mL^−1^), and DNAse I (10 μL·mL^−1^) (both Sigma‐Aldrich) for PDAC organoids and TrypLE™ Express alone for HNSCC organoids. The trypsin inhibitor from *Glycine max* was used to stop the reaction. Single‐cell suspensions were diluted 1 + 1 in growth factor‐reduced matrigel (Corning, Wiesbaden, Germany) for PDAC cells or BME (R&D Systems, Wiesbaden, Germany) for HNSCC cells, respectively. 10 μL of the cell suspension was immediately seeded into 96‐well U‐bottom plates in a range of 1–3200 cells per well. After 20 min, culture medium containing 10 μm ROCK Inhibitor Y‐27632 was added to the wells. Subsequently, cells were irradiated and incubated for 21 days. Analysis was performed as described for suspension cells.

### 2.9 Fluorescence microscopy of organoids

For fluorescence microscopy, single‐cell suspensions of one PDAC organoid line were seeded into eight‐chamber slides (Ibidi, Gräfelfing, Germany) and irradiated at the indicated doses. After 21 days, medium was removed and matrigel drops were covered with fresh medium containing 10 μg·mL^−1^ Hoechst 33342 and 20 μg·mL^−1^ propidium iodide (both from Sigma‐Aldrich). Organoids were imaged after an incubation time of 1 h at room temperature with an AxioObserver Z1 inverted microscope equipped with an EC Plan‐Neofluar 5×/0.16 Ph1 and an AxioCam MRm camera.

## 3 Results

### 3.1 Nonlinear phenomena of clonogenicity: Cellular cooperation and competition and their impact on data analysis in limiting dilution assays

To assess the impact of nonlinear phenomena on the robustness of clonogenicity analyses in the LDA format, we measured clonogenic survival upon irradiation in a panel of adherent cancer cell lines with varying degrees of cooperative and competitive clonogenic growth behavior as previously demonstrated in the CFA format [22]. For the given cell line panel, we found—as expected—that some cell lines showed nonlinear relations between the number of cells seeded (*S*) and the log‐transformed fraction of wells with clonogenic growth failure (ln(*μ*)) (Fig. 2B). This indicates that the single‐hit Poisson model, which describes a scenario where one event (i.e., clonogenic growth of one cell) occurs randomly and independently at a constant average frequency, and which is typically assumed in standard LDAs [3, 28, 29, 30, 31] is not universally applicable. To investigate this phenomenon in greater detail, we plated fixed numbers of A549 and SKLU1 single cells into multiple wells with highly different volumes of culture medium (20–5000 μL/well, 384‐well–12‐well format) and analyzed clonogenic growth. In line with the approximately linear ln(*μ*) ~ *S* relation of A549 cells, the clonogenic activity of these cells was quite stable in different culture medium volumes when plated in standard medium supplemented with 10% FBS, and the majority of wells (>80%) revealed clonogenic growth (Fig. 2C, right graph). In contrast, SKLU1 cells showed a high degree of cellular cooperation and, correspondingly, a steep decline in clonogenic growth with increasing assay volume, thus providing a demonstrative explanation for the nonlinear ln(*μ*) *~ S* relation of this cell line (Fig. 2C, left graph). These data confirm previous results showing that potentially clonogenic cells may ‘lose’ their clonogenicity if cellular cooperation via soluble factors is hampered [22]. As expected, highly variable estimates of the fraction of clonogenic cells ranging from 1/8 to 1/175 cells were obtained when applying the (obviously violated) LDA single‐hit model to SKLU1 cells in different culture medium volumes.

*Vice versa*, A549 single‐cells grew highly competitively when clonogenicity was assessed under serum‐reduced conditions, and frequency estimates of clonogenic cells covered the full range from 1/1 to 1/inf when analyzing the raw data with linear ln(*μ*) ~ *S* regression (Fig. 2D, right graph). Volume‐independent growth patterns were obtained for SKLU1 cells if EMEM, the basal medium recommended by the supplier, was replaced by DMEM/F12, a more nutrient‐rich medium supplemented with the identical percentage of 10% FBS (Fig. 2D, left graph). Taken together, these data demonstrate that clonogenic growth of single cells plated at limiting cell numbers is strongly affected by cell density and the composition of the culture medium, which together frame the biochemical context for single‐cell growth. Individual cells with—in principle—clonogenic potential may fail to give rise to clonogenic offspring under the limiting dilution conditions of the LDA format, whereas they may manage to survive clonogenically when supported by others, or when using alternative culture media containing all factors required for clonogenic growth at a sufficient concentration. Conversely, competition for growth factors and/or nutrients may impair clonogenic survival.

### 3.2 LDAcoop: Implementing nonlinear population dynamics into LDA data analysis

These deviations from linear clonogenic growth behavior have been described by various researchers [1, 15, 17, 22, 32, 33, 34], and cellular cooperation [12] as well as cellular competition [21] of mammalian cells provide biological explanations for violations of the single‐hit hypothesis of LDAs in these cell model systems. To account for distorting effects of nonlinear clonogenicity on the analysis of CFA‐derived survival data, we recently proposed a regression‐based mathematical analysis workflow CFAcoop, where the relation of clonogenic cells and seeded cells is modeled via power law [1, 22]. We now aimed to address this issue in the mathematical analysis workflow of LDA experiments. Commonly, the fraction of clonogenic cells (*p*) in a given cell population is calculated by determining the total number of cells (*S*_*λ*=1_) that need to be plated in order to obtain on average exactly one clonogenic cell (*λ* = 1), that is, exactly one colony, per well. According to the Poisson distribution, this mean of exactly one colony per well corresponds to a probability of clonogenic growth failure (i.e., no colony per well) in 1/*e* of all wells tested, which is approximately 37%. The average of clonogenic cells obviously depends on (i) an independent probability *p* of clonogenic growth for each individual cell and (ii) the total number *S* of cells seeded. In standard linear, single‐hit Poisson models (SHPM), the expected number of clonogenic cells is described by

$$\lambda_{\text{SHPM}}=p\cdot S$$

To account for non‐linear (i.e., cooperative or competitive) clonogenic growth phenomena, analogously to CFAcoop we generalized this relation via power law and included a nonlinearity parameter *b* to

$$\lambda =p\cdot S^{b}$$

**Fig. 3:**
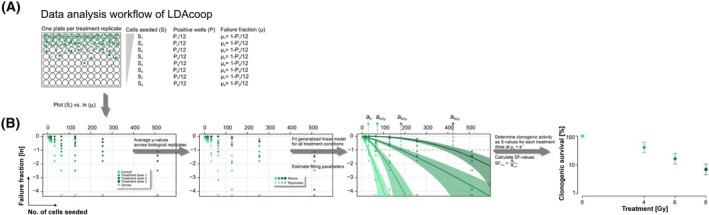
Overview of the LDA data analysis workflow with the R tool LDAcoop. (A) Layout of a representative 96‐well plate with a geometric dilution series of 8 different cell numbers (*S*) seeded in 12 technical replicates. The fraction of wells with clonogenic growth and without clonogenic growth (failure wells) is determined for each cell number. (B) Raw data of failure fractions (*μ*) of different cell numbers are plotted in an ln(*μ*) *~ S* graph. *μ*‐values are averaged, generalized linear regression models are fitted to the ln(*μ*) *~ S* data for controls and each treatment dose to determine the clonogenic activities (*S*‐values corresponding to the failure rates *μ = 1*/*e*), and the respective 95% confidence bands are displayed in pale green. Clonogenic survival curves are generated by normalizing the clonogenic activity values of each treatment dose to that of the untreated controls. Data (of BT20 cells) were collected in four independent biological replicates each performed in 12 technical replicates.

This generalized formulation allows the estimation of the number of seeded cells required to yield on average one clonogenically active cell per well, as well as the cooperativity/competitivity parameter *b*, based on the observed fraction of wells without clonogenic growth. From the fractions of clonogenic cells under control and treatment conditions, the surviving fractions (SFs) can then be inferred. A detailed description of the mathematical background and the development of the R package LDAcoop is given in the Materials and Methods section, and a step‐by‐step example of LDA raw data collection, scoring, as well as plotting of multiple replicate data, subsequent non‐linear ln(μ) *~ S* fitting, and the final clonogenic survival results is shown in Fig. 3.

In full analogy to our CFAcoop approach for CFA analysis [1, 22], the exponent *b* is determined independently for each treatment condition of an experiment by assessing the probability of clonogenic growth over a range of different cell numbers seeded in *n* replicate wells. In case of cellular cooperation, *b*‐values of >1 are observed, whereas cellular competition is characterized by *b*‐values <1. Within the cell line panel shown in Fig. 2B, we found clear deviations from the linearity assumption (*b* ≈ 1) in both directions with *b*‐values ranging from 0.70 to 0.92 for T47D cells and from 1.54 to 2.60 for SKLU1 cells at different radiation doses, which were in good agreement with the results obtained in the CFA format (Fig. S2 and [22]). Interestingly, *b*‐values trended to be higher in the CFA format. This may reflect the fact that CFAcoop analysis operates at the level of 20 detected colonies, whereas LDAcoop analysis is performed at the level of a single colony, likely resulting in stronger non‐linear growth phenomena as quantified by the *b*‐value.

**Fig. 4:**
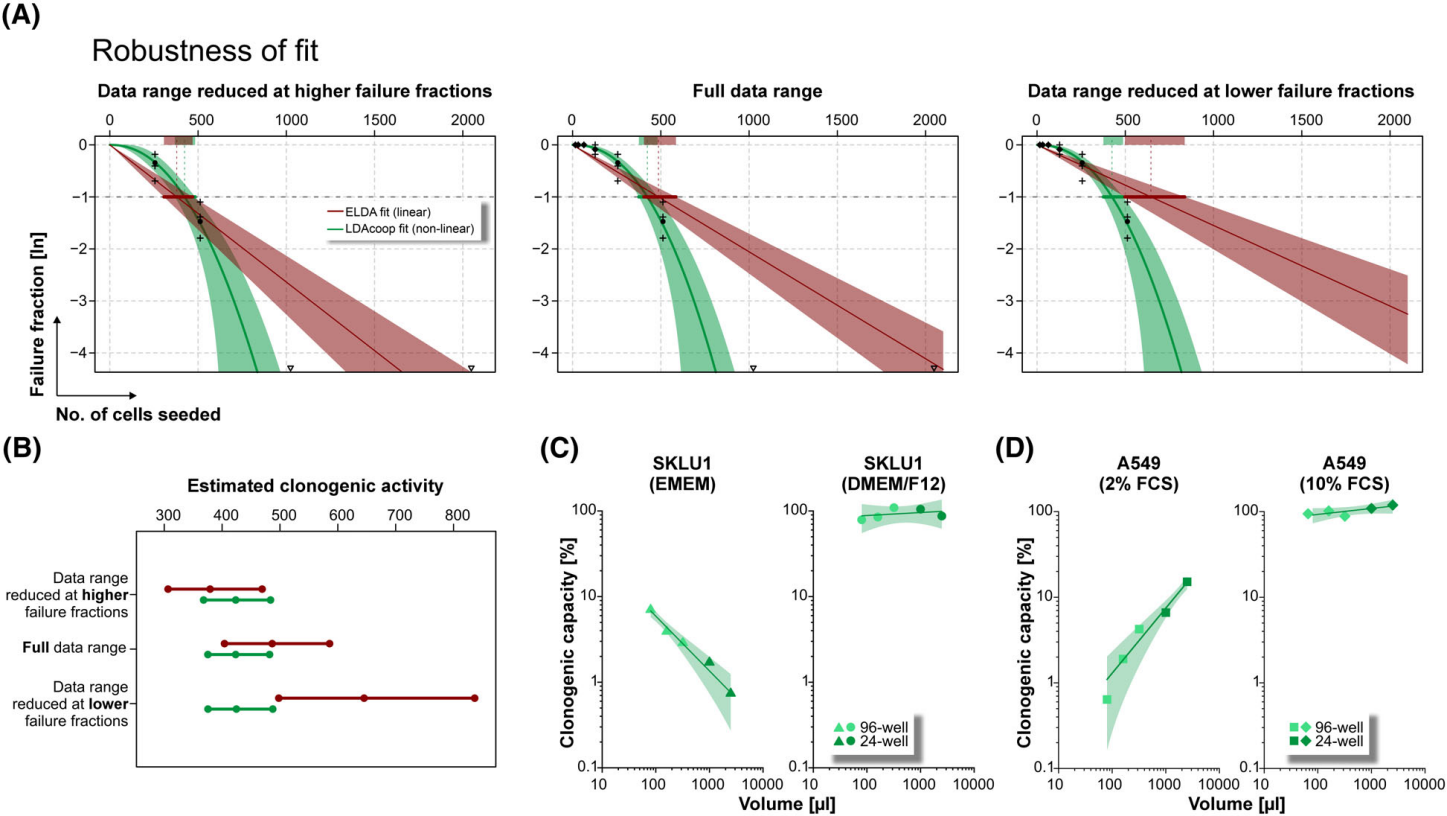
Implementation of a nonlinearity coefficient *b* strongly increases robustness of fit. (A) Fits with linear ln(*μ*) *~ S* [3] and non‐linear ln(*μ*) ~ *S* ^*b*^ regression (LDAcoop) of the full data set of BT20 cells shown in Fig. 2B (8 Gy), or reduced versions thereof with omission of data points in the higher (left panel) or lower (right panel) cell number range. Regression lines and confidence intervals are shown. (B) Estimated clonogenic activities and their confidence intervals as calculated in A. (C, D) Dependence of the clonogenic capacity on the culture conditions. LDAs of SKLU1 and A549 cells were performed in different media, assay volumes, and multi‐well plate formats in analogy to Fig. 2C,D, and data were analyzed using LDAcoop. The clonogenic capacity (i.e., the percentage of clonogenic cells within the population) is displayed as means of four biological replicates each performed in 12 technical replicates with power regression lines and 95% confidence intervals superimposed.

The implementation of the exponent *b* into the analysis workflow stabilizes the results output and increases its robustness (Fig. 4A,B). The estimated clonogenic activity of a given cell population with nonlinear clonogenicity as well as the corresponding confidence intervals remain very stable, irrespective of whether the full available data set or reduced versions thereof (data set reduced at the higher or lower range of seeded cells) are used for calculation. In strong contrast, utilizing the linear analysis approach [3] yields highly varying results for the estimated clonogenic activity, including the corresponding confidence intervals. Hence, power law‐based modeling of nonlinear clonogenic growth phenomena as implemented in LDAcoop clearly outperforms LDA analysis tools that rely on linear equations in conditions with violated linearity assumption. This was further supported by deviance and Akaike information criterion (AIC) analyses, in which LDAcoop yielded lower deviance in all datasets and lower AIC values in nearly all cases, demonstrating a systematically better goodness‐of‐fit than the linear LDA model. In several datasets, the improvement in fit was also statistically significant based on likelihood‐ratio tests (LRT, Table S4). While the original authors explicitly discouraged application of the linear LDA model outside the method's valid scope [3], such cases remain common in practice.

Taking advantage of the LDAcoop tool, we analyzed the dependence of the clonogenic capacity of A549 and SKLU1 cells on the respective culture conditions. Dilution series of both cell lines were seeded, analogous to the experiments shown in Fig. 2, using different culture media, culture volumes, and multiwell plate formats. For the cooperatively growing SKLU1 cells, clonogenic capacity ranged from >7% to <1% with increasing assay volume in EMEM but remained largely stable at >80% in DMEM/F12 (Fig. 4C). Conversely, owing to their competitive growth behavior, the clonogenic capacity of A549 cells decreased from >15% to <1% with decreasing assay volume in 2% FBS, whereas it stayed virtually unchanged at >80% in 10% FBS (Fig. 4D). These condition‐dependent variations thus reflect genuine differences in cellular cooperation and competition across distinct biochemical contexts, which—assuming single‐cell independence—are not adequately captured by linear LDA analysis algorithms.

### 3.3 Benchmarking LDA and CFA: LDA Is superior for complex culture models and applications with higher throughput requirements

**Fig. 5:**
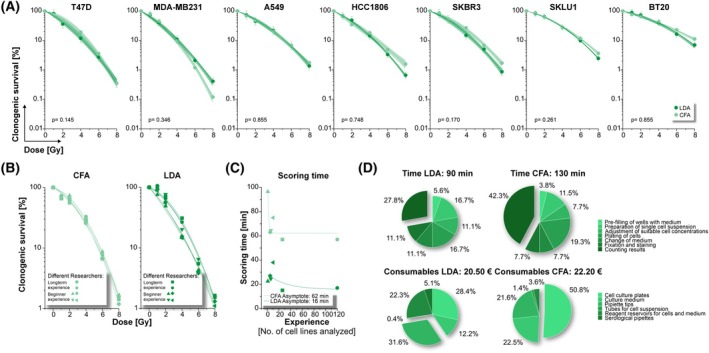
Clonogenic survival results generated by CFA and LDA protocols are highly similar, but the LDA format requires less time for scoring. (A) Comparison of the survival curves of seven cancer cell lines generated by CFA and LDA protocols. CFA data were measured in three (T47D and HCC1806 cells) or four (all other cell lines) independent biological replicates, subjected to analysis and CFAcoop, and taken from Brix et al. [22]. LDA data were acquired in three (SKBR3 and A549 cells) or four (all other cell lines) independent biological replicates, and clonogenic survival curves were calculated using the R tool LDAcoop. Means of independent biological replicates ±95% confidence intervals as obtained from CFAcoop and LDAcoop are shown with linear‐quadratic regression lines superimposed and shaded areas depicting 95% confidence intervals of the respective fits. Comparisons between data sets were performed using two‐way ANOVA with *treatment* and *method* as factors. *P*‐values for the factor *method* are reported. (B) Comparison of inter‐researcher variability in the CFA and LDA format. Survival data of each one CFA and one LDA experimental replicate of SKBR3 cells were scored by six independent researchers with varying extents of experience and subjected to downstream analysis with CFAcoop or LDAcoop, respectively. (C) Time required for scoring the data shown in (A). (D) Comparison of total experimentation time and costs of CFA and LDA.

Considering the essentially identical readouts of CFA and LDA—both are used to determine the fraction of clonogenic cells after various treatments—we set out to compare the results generated by the two methods. Clonogenic survival curves for the seven cell lines were calculated by using LDAcoop, and the results were compared to previously generated CFAcoop results from cells of the same cultures (difference of no more than 3 culture passages between data collection) [22]. The survival curves of the seven cell lines were highly similar (Fig. 5A). Obviously, both CFA and LDA can be used equivalently to assess clonogenic survival, provided that nonlinear clonogenic growth behavior is properly considered.

Given the congruence of CFA and LDA results outputs, we next benchmarked the two methodologies against each other with regard to time and costs. Six independent researchers with different levels of experience in clonogenic assay analysis were asked to score identical sets of CFA and LDA plates with each of six different treatment conditions. While highly similar survival results were obtained for the CFA format, researchers with less experimental experience tended to underscore the LDA format, particularly in the lower dose range of treatment (Fig. 5B). However, the time required for assay scoring was significantly shorter for the LDA format—irrespective of the researchers' level of experience (Fig. 5C), translating into a total reduction of time needed for an entire LDA experiment compared to the CFA format. The costs for consumables of both methods were comparable (Fig. 5D).

**Fig. 6:**
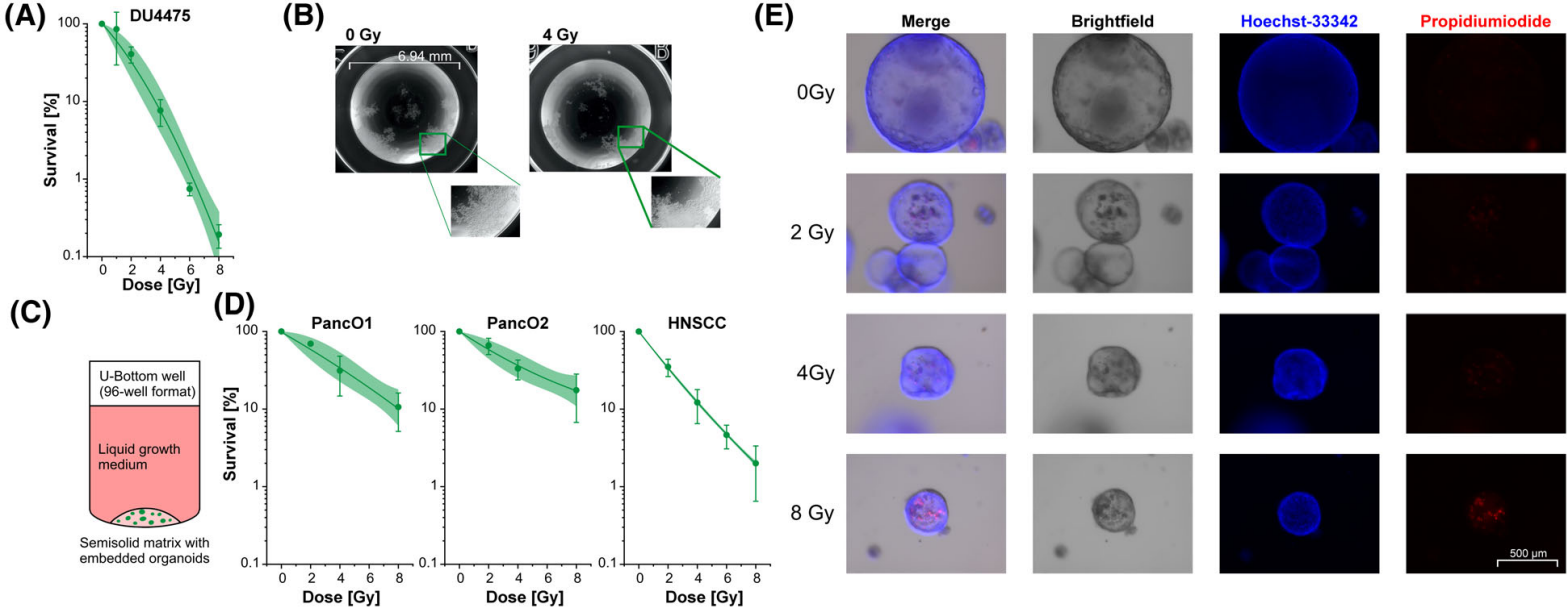
Clonogenic survival analyses of non‐adherently growing cell lines and organoids using the LDA format. (A) Analysis of clonogenic survival upon irradiation by LDAcoop using the non‐adherently growing breast cancer cell line DU4475. Means ± 95% confidence intervals of survival estimates of three independent biological replicates as obtained from LDAcoop are shown. Linear‐quadratic regression is superimposed with shaded areas indicating 95% confidence intervals of the respective fit. (B) Photographs of untreated (left) and irradiated (right) DU4475 cells on the day of analysis (d21 after plating). One representative well of a 96‐well plate is shown in the large photograph (surface area = 0.32 cm^2^). Inlays depict colony growth at higher magnification. (C) Scheme depicting clonogenic organoid formation in the 96‐well format. (D) Analysis of treatment response of patient‐derived organoids by LDA. Single‐cell suspensions of two pancreatic cancer organoids (PancO1, PancO2) and one organoid from a head and neck squamous cell carcinoma (HNSCC) were irradiated at 0–8 Gy and clonogenic organoid formation was analyzed by LDAcoop. Means and 95% confidence intervals boundaries of three independent biological replicates are shown. Linear‐quadratic regression is superimposed with shaded areas indicating 95% confidence intervals of the fit. (E) Transmitted light and fluorescence microscopy images of representative pancreatic cancer organoids on the day of analysis (d21 after plating and irradiation at the indicated dose). Live‐dead staining of matrigel‐embedded organoids was performed with Hoechst 33342 and propidium iodide. Scale bar in bottom right image (500 μm) applies to all panels of subfigure E.

Considering the advantages of clonogenic survival analyses in the LDA format in 2D settings with adherent cells, the performance of LDAcoop was tested in cell culture models for which CFA protocols are highly challenging and time‐consuming to employ. We used the suspension breast cancer cell line DU4475, which is not able to form adherent colonies in liquid culture medium [35] and analyzed its survival upon irradiation by LDA. While the determination of the precise colony count/well in liquid culture media would obviously be impossible and thus require more labor‐intensive surrogate analyses in semisolid 3D matrices, LDA scoring was well feasible even without fixation and staining of the cells (Fig. 6A,B). With *b*‐values of up to 3.75, cellular cooperation in DU4475 cells was profound, thus confirming the necessity to consider this phenomenon in LDA analysis.

We also examined clonogenic survival upon irradiation in three different patient‐derived organoid cell lines which were cultured in 3D matrices covered by liquid culture medium (Fig. 6C). Organoid formation by irradiated single cells seeded in the LDA format could be obtained in individual wells for all conditions tested, and viability of organoids was confirmed in selected culture wells by immunofluorescence microscopy (Fig. 6D,E). These data show that the LDA format can be easily adapted to challenging cell culture models, whereas the necessity to determine the precise number of colonies in individual wells frequently obstructs the applicability of the CFA format, particularly if non‐adherent cells or organoid culture systems are analyzed.

**Fig. 7:**
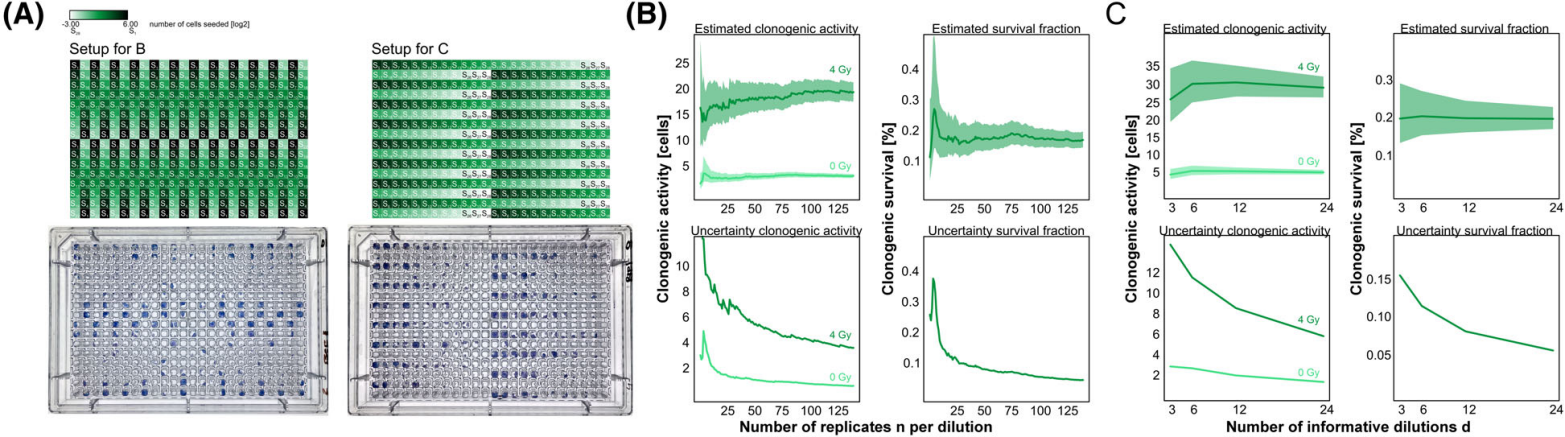
Upscaling potential of the LDA protocol to the 384‐well format and optimization of assay design. (A) Layout of 384‐well plates to analyze the impact of varying numbers of technical replicates *n* (left panel) or varying numbers of informative cell dilutions *d* (right panel) on the robustness of results output. Photographs of HCC1806 cells grown in the respective 384‐well formats. Data were acquired in three independent biological replicates. (B) Analysis of clonogenic activity, clonogenic survival, and the corresponding uncertainties (width of confidence interval) of the setup with varying numbers of technical replicates *n* (*n* = 6 to *n* = 144) as shown in (A). (C) Analysis of clonogenic activity, clonogenic survival, and the corresponding uncertainties (width of confidence interval) of the setup with varying numbers of informative cell dilutions *d* as shown in (A).

Finally, we made use of 384‐well culture dishes to interrogate the upscaling potential of the LDA format. Yes/no scoring of culture plates was well feasible, and the increase in information density as compared to 96‐well dishes of identical size was fourfold (Fig. 7A). Additionally, increasing the number of technical replicates *n* (Fig. 7B) per cell density as well as increasing the number of informative dilutions *d* (Fig. 7C) can be instrumentalized to generate more reliable results with smaller confidence intervals. Hence, these experiments demonstrate the potential for larger‐scale screening approaches in the LDA format, which would be much more challenging in the CFA format.

## 4 Discussion

In the present study, we developed a mathematical modeling approach that integrates nonlinear phenomena of clonogenicity into LDA analysis. We introduce LDAcoop—a computational LDA analysis tool that accounts for cooperative and competitive clonogenic growth behavior in analogy to CFAcoop in which power law‐based analysis of CFA data captures nonlinear clonogenicity [1]. LDAcoop is provided in an R package version (https://cran.r‐project.org/web/packages/LDAcoop/index.html) as well as in an online shiny app format (https://helmholtz‐munich‐zyto.shinyapps.io/LDAcoop/).

The clonogenic capacity of single cells can be modulated by soluble factors, which are fully absent or present at limiting concentrations in the culture medium. In case of cellular cooperation, higher single‐cell densities result in elevated concentrations of cell‐derived factors, finally leading to increased clonogenicity [1, 22]. *Vice versa*, cellular competition for growth‐limiting nutrients and/or growth factors or driven by growth‐inhibiting factors may occur, respectively [18]. In consequence, the pattern of auto‐ and/or paracrine growth modulation of single cells is shaped by (i) the secretome of the cells, (ii) the biochemical composition of the culture medium, (iii) the kinetics of metabolite‐receptor interactions, and (iv) the biophysical properties of the culture system [1, 22, 36]. This also implies that a mammalian cell's clonogenic capacity is not an inherent yes/no characteristic but is rather contingent upon the permissiveness of the chosen culture conditions.

Cell density effects under limiting dilution conditions have been extensively characterized *in vitro*. Historically, mammalian single‐cell growth experiments were experienced to be very challenging because unconditioned, cell‐derived growth factor‐free culture media were recurrently observed to be insufficient for sustaining single‐cell growth [5, 6], thus giving rise to the concept of ‘autocrine secretion’ by Sporn and Todaro [11]. More recently, we and others demonstrated that soluble cell‐derived factors enforce clonogenic survival and proliferation rates even when using modern, advanced culture media [15, 22, 37]. Mathematical modeling as well as experimental evidence suggests that autocrine growth stimulation may even be relevant in scenarios where only one singular cell is seeded into a culture dish [36]. We conclude that a strict binary categorization of individual cells of a given population into clonogenic versus nonclonogenic cells is a misleading oversimplification. It can distort the calculation of clonogenic cell frequencies because even cells with high clonogenic potential may show impaired clonogenicity or complete abrogation of their clonogenic capacity, respectively, if the cells' culture requirements are insufficiently addressed. Along the same lines, abrupt deprivation of individual cells from cooperating neighbors has been proposed as an explanation for low plating efficiencies in clonogenic *in vitro* experiments, slow recovery of cell lines when passaging them at limiting cell densities, and poor success rates in the establishment of stably growing tumor cell lines from *ex vivo* preparations and clinical tumor specimens [38], all of which can be interpreted as examples of the Allee effect of cell populations growing under low densities *in vitro* [14, 16].

Because it is technically impossible to find ‘ideal’ culture conditions, which fully compensate for cooperative and competitive effects in clonogenic survival experiments for each cell model system and each treatment condition of interest, we recently proposed a mathematical approach that accounts for these phenomena in downstream CFA data analysis [1, 22]. In CFAcoop, the relation between the number of single cells seeded (*S*) and the number of colonies obtained (*C*) is fitted by power law according to *C = a × S* ^*b*^ [1, 22]. In the present study, we transferred this approach to the analysis of clonogenic survival in the LDA format and developed the R package LDAcoop. In full analogy to CFAcoop, the degree of nonlinear clonogenicity in LDAcoop is represented by the exponent *b*, which is mathematically equivalent to the exponent used in CFAcoop [1]. LDAcoop allows nonlinear relations between *S* and ln(*μ*), which we indeed observed in various cell lines as indicated by *b*‐values ranging from 0.7 (in case of moderate cellular competition) to 3.75 (for extreme cellular cooperation). A central assumption of this framework is that wells are seeded with the same expected number of cells, which is a common simplification in limiting dilution analysis. While actual cell numbers may vary in practice, explicitly modeling this overdispersion would require alternative frameworks and is beyond the scope of the present work.

Alternative nonlinear LDA quantification approaches have already been proposed. Bonnefoix et al. focused on alternative statistical testing procedures for goodness‐of‐fit assessment, moving away from the mechanistic SHPM formulation [39]. Ellison et al. extended the SHPM by introducing a Gamma‐Poisson formulation to account for overdispersion in lambda and further embedded this into a multi‐level mixed‐effects framework [40]. While this also nests the classical SHPM as a special case, the formulation departs conceptually from the mechanistic interpretation of clonogenic cooperation and competition and requires specialized solvers. In contrast, LDAcoop retains the mechanistic structure of the SHPM, providing a direct generalization through a power‐law relation that captures cooperative (*b* > 1) and competitive (0 < *b* < 1) dynamics and can be estimated within a standard generalized linear model (GLM) framework. Nevertheless, linear models to date clearly dominate routine LDA analysis [3]. Against this background, we now present a mathematical generalization of the most widely used linear model, which additionally captures the non‐linear cooperative and competitive dynamics.

Of note, LDAcoop differs from linear LDA analysis tools with regard to the mathematical information that is inferred from experiments where all‐negative responses occur for one or more cell densities: For LDAcoop, an all‐negative response resulting from seeding each *S* cells into *n* wells is not equivalent to seeding each *N* cells into *s* wells with the same all‐negative result, because the dilution of cell‐derived factors as well as the total amount of medium‐derived factors per cell differ between the two settings if *S* ≠ *n*. In contrast, linear, single‐hit models ignore the impact of initial single‐cell density and calculate identical estimates of clonogenic cell frequencies in these two scenarios, because a constant total number of cells, *S* × *n*, were analyzed [3]. The association of parameters *p* and *b* implies that LDAcoop does not provide estimates of clonogenic cell frequency if not a single well exhibits clonogenic growth at a given treatment condition. We therefore recommend testing geometric series of single‐cell density dilutions to ideally obtain response patterns that cover the full range from all‐positive to all‐negative for each treatment condition of interest. In contrast, multiple cell densities with all‐negative or all‐positive responses add little information to the overall dose–response curve.

Apart from the LDA methodology, CFA protocols are routinely employed to quantify clonogenic growth of cancer cells *in vitro*. In the present study, we show that LDA and CFA formats generate highly similar survival data, provided that the impact of nonlinear cooperative and competitive clonogenic growth phenomena are properly integrated into the mathematical analysis of raw data [12, 21]. Since we demonstrated the equivalence of LDA and CFA results in various cancer cell lines, we benchmarked the two methodologies against each other and found that LDA protocols outperform CFA analyses under certain conditions. This is most obvious in settings where suspension cells or organoids are analyzed. Here, the requirement of the CFA to determine an exact colony count in a 3D matrix complicates the analysis, whereas binary scoring of the LDA format is much more rapid and also feasible for suspension cells without embedding. Hence, researchers who so far have predominantly relied on CFA‐based approaches may consider the LDA a time‐efficient and widely accepted alternative for analyzing clonogenic single‐cell growth. Moreover, the limited applicability of the CFA format to patient‐derived organoids, suspension cells, and higher throughput applications represents a major bottleneck for integrating clonogenic survival as an experimental endpoint in larger‐scale screening approaches and drug sensitivity testing using complex cancer cell models. These limitations can be overcome by employing the LDA format.

A major aspect in the design of LDA experiments is to find an adequate number of technical replicates for each cell density. Most experiments performed in the present study rely on technical replication of *n* = 12 and *d* = 8 different single‐cell dilutions tested per treatment, that is, 96 wells for each treatment condition. More robust results with reduced uncertainties can be obtained by increasing the number of controls which serve for data normalization (e.g., by using 2 × 96 wells for the untreated controls). Additionally, both higher‐resolution data sets with reduced uncertainties at the costs of higher technical replication (e.g., *n* = 48, *d* = 8) or, alternatively, lower‐resolution data sets with multiple treatments and/or different cell lines at the costs of larger uncertainties (e.g., *n* = 8, *d* = 8 for six different cell lines seeded on one plate) can be acquired with a fixed number of total wells. Irrespective of the desired assay format, 384‐well plates allow experimental upscaling as compared to 96‐well plates since the density of information per surface area is increased by 4‐fold. Albeit limited by a maximal culture medium volume of 10–12 μL per well, which might escalate the impact of cellular competition at rather low cell densities, even automated LDA analysis on 1536‐well plates could be of interest for specific research questions, including large‐scale screenings of therapy resistance and/or drug response relationships, with the observation endpoint being clonogenic survival.

Cooperation and competition, which we now have implemented into LDA data analysis, should not be considered artificial *in vitro* phenomena of mammalian cell culture. Although beyond the scope of this study, there is clear evidence that both also occur *in vivo*. For instance, soluble factors such as Decapentaplegic (DPP) in drosophila wings [41] or Wingless/Integrated (WNT) antagonists in murine intestinal crypts [42] are prominent examples of cellular competition mediated by soluble factors. *Vice versa*, cooperating subclones have been identified within established tumors [43, 44, 45]. While most commonly applied *in vitro*, the LDA format has been frequently used *in vivo*, for example, to assess tumor outgrowth following injection of defined cell numbers [46]. This scenario is conceptually similar to metastatic dissemination, where single tumor cells attempt to colonize distinct organ microenvironments that may or may not support clonogenic outgrowth. In such contexts, non‐linear clonogenicity phenomena reflecting cooperative growth and microenvironmental dependencies are likely to be even more pronounced [47, 48, 49, 50]. By explicitly modeling these non‐linearities, LDAcoop can thus increase the robustness and interpretability of both *in vitro* and *in vivo* LDAs.

## 5 Conclusions

In conclusion, our study presents a mathematical framework that enhances the analysis of clonogenic survival data acquired in the LDA format by incorporating nonlinear, cooperative and competitive growth phenomena and introduces LDAcoop, an R package including an online shiny app version thereof. In view of the high congruence of clonogenic survival results obtained by CFA and LDA protocols, the superiority of the LDA format in terms of scoring speed, applicability in suspension and 3D organoid culture settings, as well as its upscaling potential positions LDAcoop as a valuable tool for larger‐scale pharmacogenomic screenings and automated analyses of therapy resistance and drug response with the observation endpoint clonogenic survival, thus resolving significant bottlenecks in research fields which currently rely predominantly on the CFA format.

## Conflict of interest

The authors declare no conflict of interest.

## Author contributions

KL, NB, and HZ were involved in conceptualization and study design. NB, DS, and KL were involved in data curation. DS, KL, and NB were involved in formal analysis. KL, HZ, MS, CB, and NB were involved in funding acquisition. NB, KG, and GD were involved in investigation. NB, DS, and KL were involved in methodology. KL, CB, HZ, DS, BD, MS, AA, GB, UM, and JM were involved in resources. DS and BD were involved in software. KL, HZ, and MS were involved in supervision. NB, DS, and KL were involved in validation. NB, KL, and DS were involved in writing (original draft) and writing (review and editing).

## Supporting information


**Fig. S1.** Methods of uncertainty estimation implemented in LDAcoop.
**Fig. S2.** Comparison of the non‐linearity coefficients b of clonogenic growth behavior as determined in the CFA and LDA format.
**Table S1.** Overview of cell culture media used for adherent and suspension cells.
**Table S2.** Formulations of PDAC organoid media.
**Table S3.** Formulation of HNSCC organoid media.
**Table S4.** Comparison of model fits between the classical limiting dilution analysis (LDA, slope fixed to 1) and the generalized LDAcoop model (slope free).

## Data Availability

All raw data from the limiting dilution assays shown in Figs 2B–D, 5A,B, 6A,D, along with the original code, are publicly available as part of the R package LDAccop (https://cran.r‐project.org/web/packages/LDAcoop/index.html) or as part of the online shiny app version LDAcoop (https://helmholtz‐munich‐zyto.shinyapps.io/LDAcoop/), respectively. Microscopy data depicted in Fig. 6E will be shared by the corresponding author upon request. Any further information needed to reanalyze the data presented in this paper can be obtained from the corresponding author upon request.
