# Supplementary material for: LDAcoop: Integrating non‐linear population dynamics into the analysis of clonogenic growth in vitro
Source: Mol Oncol. 2025 Dec 26;20(5):1237–52. doi: 10.1002/1878-0261.70185 (PMC13155136; doi:10.1002/1878-0261.70185)
Supplement: Supplementary file 1 — Fig. S1. Methods of uncertainty estimation implemented in LDAcoop. Fig. S2. Comparison of the non‐linearity coefficients b of clonogenic growth behavior as determined in the CFA and LDA format. Table S1. Overview of cell culture media used for adherent and suspension cells. Table S2. Formulations of PDAC organoid media. Table S3. Formulation of HNSCC organoid media. Table S4. Comparison of model fits between the classical limiting dilution analysis (LDA, slope fixed to 1) and the generalized LDAcoop model (slope free). [file MOL2-20-1237-s001.pdf]

Supplemental Tables, Figures, and References

Cell line	Identifier	Supplier	Culture medium	FCS [%]	CO_2 [%]
T47D	RRID:CVCL_0553 Cat#300353	CLS	RPMI 1640 (GlutaMAX™ + 35 mM HEPES) + P/S	10	5
SKBR3	RRID:CVCL_0033 Cat#300333	CLS	DMEM (high glucose, GlutaMAX™, pyruvate) + P/S	10	7.5
BT20	RRID:CVCL_0178 Cat#300130	CLS	DMEM/F-12 (1 + 1, GlutaMAX™) + P/S	10	7.5
MDA-MB231	RRID:CVCL_0062 Cat#HTB-26	ATCC	DMEM (high glucose, GlutaMAX™, pyruvate) + P/S	10	7.5
HCC1806	RRID:CVCL_1258 Cat#CRL-2335	ATCC	RPMI 1640 (GlutaMAX™ + 35 mM HEPES) + P/S	10	5
DU4475	RRID:CVCL_1183 Cat#ACC427	DSMZ	RPMI 1640 (GlutaMAX™ + 35 mM HEPES) + P/S	20	5
A549	RRID:CVCL_0023 Cat#ACC107	DSMZ	DMEM (high glucose, GlutaMAX™) + P/S	10	7.5
SKLU1	RRID:CVCL_0629 Cat#300335	CLS	EMEM basic medium + P/S	10	5

Supplemental Table 1: Overview of cell culture media used for adherent and suspension cells. "P/S" corresponds to 100 U/mL penicillin and 0.1 mg/mL streptomycin.

Base Medium	Supplier	
Advanced DMEM/F-12	Thermo Fisher 12634010	
HEPES	Sigma H 0887	1%
GlutaMAX	Thermo Fisher 35050061	1%
Primocin	Invivo Gen ant-pm-05	100 µg/ml
**Reagent**	**Supplier**	**Concentration**
Base medium		35.5%
WNT3a conditioned medium		50%
R-spondin conditioned medium		10%
Nicotinamide	Sigma-Aldrich N 0636	10 mM
B27 Supplement	Thermo Fisher 17504044	1x
Human FGF10	PeproTech 100-26	100 ng/ml
Human EGF	PeproTech AF-100-15	50 ng/ml
Murine Noggin	PEPRO 120-10C-20	100 ng/ml
A83-01	Sigma-Aldrich SML0788	0.5 µM
Prostaglandin E2	Tocris TOC 2296/10	1 µM
Human Gastrin	Sigma-Aldrich G9020	10 nM
N-acetyl-L-cysteine	Sigma-Aldrich A9165	1.25 mM

Supplemental Table 2: Formulations of PDAC organoid media according to [26].

Base Medium	Supplier	
Keratinocyte SFM	Thermo Fisher Scientific 17005042	
Reagent	Supplier	Concentration
Bovine Pituitary Extract	Thermo Fisher Scientific 17005042	50 µg/ml
Human EGF	Thermo Fisher Scientific 17005042	1 ng/ml
$CaCl_2$	Sigma-Aldrich C5080	0.6 mM
Primocin	Invivo Gen ant-pm-05	100 µg/ml

Supplemental Table 3: Formulation of HNSCC organoid media according to [27].

**Comparison of model fits for data shown in Figure 2B**								
		T47D	MDA-MB231	A549	HCC1806	SKBR3	SKLU1	BT20
**0 Gy**	**D_LDA**	79.588	24.032	33.170	34.246	13.537	24.502	29.653
	**D_LDAcoop**	60.763	23.524	33.103	33.143	12.360	12.129	27.554
	**p**	< 0.001	0.476	0.796	0.294	0.278	< 0.001	0.147
	**AIC_LDA**	146.798	69.418	66.896	91.885	48.233	48.428	84.616
	**AIC_LDAcoop**	129.973	70.910	68.829	92.781	49.055	38.055	84.517
**8 Gy**	**D_LDA**	68.279	54.836	29.779	50.255	24.927	60.472	58.090
	**D_LDAcoop**	65.905	49.376	29.747	38.554	22.753	27.655	10.758
	**p**	0.123	0.019	0.858	< 0.001	0.140	< 0.001	< 0.001
	**AIC_LDA**	125.316	114.553	69.394	89.510	63.698	87.012	87.510
	**AIC_LDAcoop**	124.941	111.092	71.362	79.810	63.524	56.195	42.179

**Comparison of model fits for data shown in Figure 4A-B**				
		Data range reduced at higher failure fractions	Full data range	Data range reduced at lower failure fractions
**0 Gy**	**D_LDA**	39.558	58.090	20.907
	**D_LDAcoop**	10.695	10.758	5.821
	**p**	< 0.001	< 0.001	< 0.001
	**AIC_LDA**	68.979	87.510	44.065
	**AIC_LDAcoop**	42.115	42.179	30.979

Supplemental Table 4: Comparison of model fits between the classical limiting dilution analysis (LDA, slope fixed to 1) and the generalized LDAcoop model (slope free). Deviance (D) values are reported for both models. Improvement in nested model fits was formally tested using likelihood-ratio-tests (LRT). AIC was calculated to evaluate model adequacy.

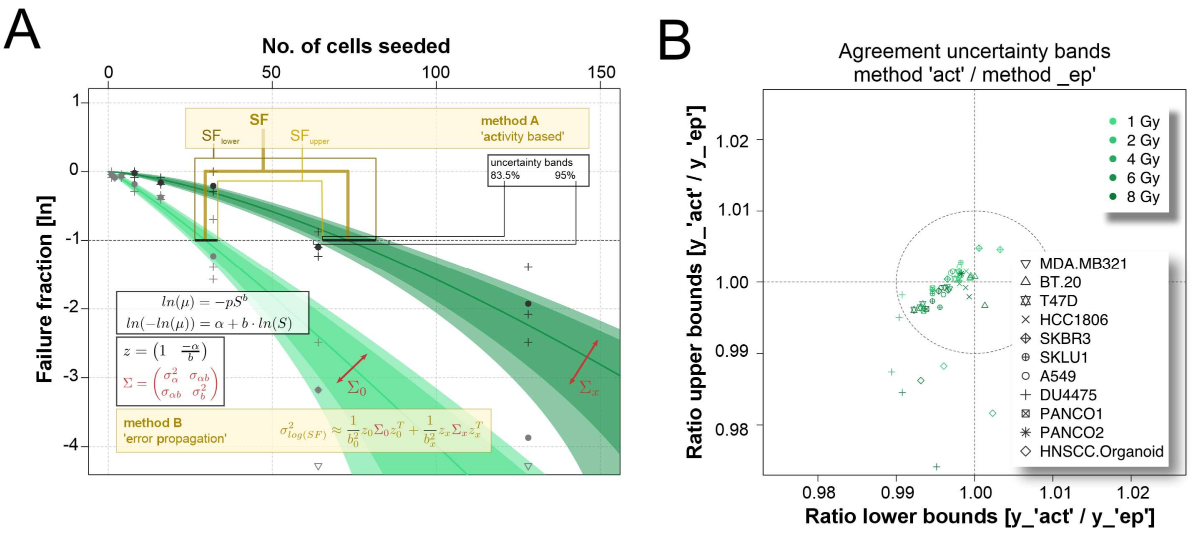

Supplemental Figure 1: Methods of uncertainty estimation implemented in LDAcoop A) Uncertainties of surviving fractions as estimated based on activity (method A) as described in the Materials and Methods section or via error propagation (method B). Both methods are available in LDAcoop. **B)** Comparison of uncertainty bands obtained via method A and method B demonstrates high agreement of both methods. The ratio [uncertainties obtained by method A / uncertainties obtained by method B] of both lower(x-axis) and upper bound (y-axis) are depicted.

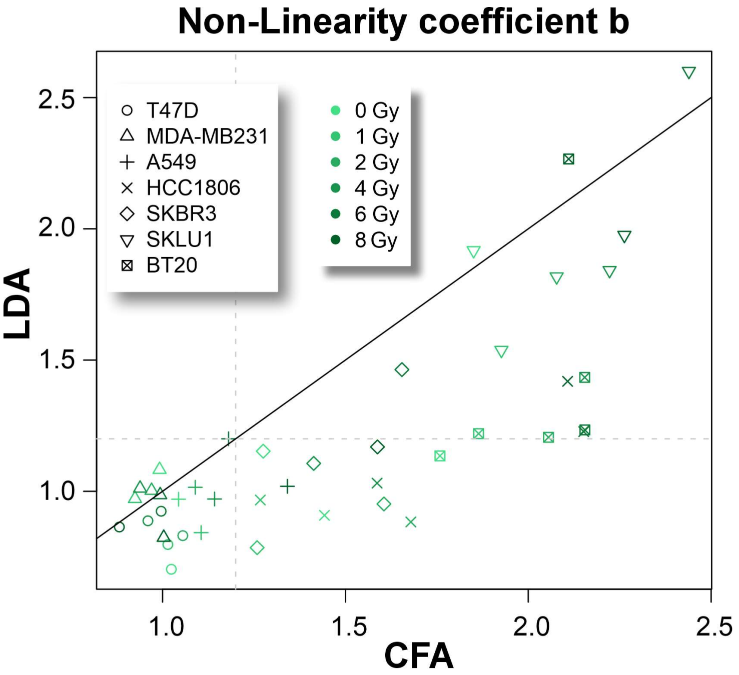

Supplemental Figure 2: Comparison of the non-linearity coefficients b of clonogenic growth behavior as determined in the CFA and LDA format. Non-linearity coefficients of all cell lines were determined at different treatment conditions in the CFA and LDA format and calculated via CFAcoop and LDAcoop, respectively. Both CFA and LDA data of all cell lines were generated in three to four independent biological replicates of which mean b-value pairs ($b_{CFA} | b_{LDA}$) are displayed for each radiation dose.
